# Sex differences in risk factors, stroke etiology, treatment characteristics, and outcomes after endovascular treatment for anterior circulation ischemic stroke

**DOI:** 10.3389/fstro.2026.1946990

**Published:** 2026-09-24

**Authors:** Marieta Peycheva, Charlotte Berger, Marino Costa, Lee Siegenthaler, Fiona Huber, Ismaël Rizzo, Marina Krasteva, Morin Beyeler, Tatiana Bremova-Ertl, Nedelina Slavova, Pasquale Mordasini, Roza M. Umarova, Philipp Bücke, Kateryna Antonenko, Angelika Hoffmann, Sara Pilgram-Pastor, Mirjam R. Heldner

**Affiliations:** 1 Department of Neurology, University Hospital “Saint George”, Medical University Plovdiv, Plovdiv, Bulgaria; 2 Department of Neurology, Inselspital, University Hospital and University of Bern, Bern, Switzerland; 3 Department of Neurology, University Children's Hospital, Sofia, Bulgaria; 4 Department of Neuroradiologie, Kantonsspital Aarau, Aarau, Switzerland; 5 Institute of Diagnostic and Interventional Neuroradiology, Inselspital, University Hospital and University of Bern, Bern, Switzerland

**Keywords:** acute ischemic stroke, anterior circulation, endovascular treatment, sex-specific differences, stroke outcomes

## Abstract

**Background and aims:**

Sex-specific differences in vascular risk factors, stroke etiology, and outcomes after endovascular treatment (EVT) remain uncertain. We investigated these differences in a contemporary single-center cohort.

**Methods:**

Consecutive patients with acute anterior circulation ischemic stroke undergoing EVT (with-/out intravenous thrombolysis) between February 2015 and March 2023 were included. Sex differences in risk factors, treatment, successful reperfusion, and 3-month outcomes (mRS ≤ 1, ≤ 2, and mortality) were examined; multivariable models included interactions with age, stroke etiology, and bridging therapy.

**Results:**

Among 1,764 patients (median age 76 years, 48% women), women were older (79.4 vs. 73.3 years, *p* < 0.001) and more often had atrial fibrillation and chronic kidney disease, while men more frequently had coronary heart disease, diabetes, and smoking history. Cardioembolic stroke was more common in women, whereas large artery atherosclerosis was more frequent in men. Successful reperfusion rates did not differ by sex (overall: 86.2%, *p* = 0.084). At 3 months, men were more likely to achieve favorable functional outcome (mRS ≤ 2: 47.6% vs. 37.5%, *p* < 0.001) and excellent functional outcome (mRS ≤ 1: 31.4% vs. 25.6%, *p* = 0.008), while mortality was higher in women (31.9 vs. 24.9%, *p* = 0.001). Female sex was not independently associated with successful reperfusion (aOR: 0.79; 95%CI: 0.60–1.06), favorable outcome (0.88; 0.69–1.11), excellent outcome (0.93; 0.73–1.19), or mortality (1.06; 0.83–1.35). A sex-by-age interaction was observed for favorable outcome (*p* interaction = 0.006), with no evidence of interaction by stroke etiology or bridging therapy.

**Conclusion:**

Women and men differ in risk profiles, stroke mechanisms, and unadjusted outcomes after EVT. However, sex was not independently associated with outcomes overall, although age may modify its association with favorable functional outcome.

## Introduction

Acute ischemic stroke (AIS) remains a leading cause of death and long-term disability worldwide (GBD 2021 Stroke Risk Factor Collaborators, 2024). For patients with anterior circulation large-vessel occlusion, endovascular treatment (EVT), with or without intravenous thrombolysis (IVT), is the established standard of care and substantially improves clinical outcomes when performed without delay (Turc et al., 2023). Growing evidence indicates that sex influences vascular risk profiles, stroke etiology, clinical presentation, treatment pathways, and recovery after AIS, highlighting the importance of understanding sex-specific determinants of outcomes after EVT (Bushnell C. D. et al., 2018; Bushnell C. et al., 2018; Grimes et al., 2024).

Despite increasing recognition of sex-related differences in acute ischemic stroke, important knowledge gaps remain (Bushnell C. D. et al., 2018; Peters et al., 2020; Swahn et al., 2024; Peycheva et al., 2024; Momen et al., 2023). Many previous studies lacked detailed sex-specific analyses of treatment strategies and procedural characteristics. In addition, several cohorts combined anterior and posterior circulation strokes or did not adequately adjust for key prognostic factors such as pre-stroke disability. Furthermore, evolving EVT techniques and changes in clinical practice over time may have contributed to the heterogeneous and sometimes conflicting findings regarding sex-specific outcomes after EVT (de Ridder et al., 2016; Dmytriw et al., 2021; Chalos et al., 2019; Madsen et al., 2019a; Dula et al., 2020; Sheth et al., 2019; Ouyang et al., 2023; Fifi et al., 2023; Single et al., 2025).

To address these knowledge gaps, we analyzed a large contemporary single-center cohort of consecutive patients with anterior circulation AIS treated with EVT, with or without IVT. We comprehensively examined sex-specific differences in baseline characteristics, vascular risk factors, stroke etiology, workflow metrics, treatment strategies, procedural characteristics, and outcomes. Furthermore, we assessed whether observed differences in outcomes persisted after adjustment for relevant baseline characteristics and treatment-related factors. By identifying determinants of outcome beyond sex alone, our findings may help inform more individualized and equitable stroke care.

## Methods

### Patients

We conducted a retrospective analysis of consecutive patients recorded in the prospectively maintained local registry of the Swiss Stroke Registry. Eligible patients presented with anterior circulation AIS and underwent EVT between February 2015 and March 2023. Patients were admitted either directly to our comprehensive stroke center or transferred from referring hospitals using a drip-and-ship model. Patients treated with IVT alone, those with posterior circulation stroke, patients without angiographically confirmed vessel occlusion, or those with missing treatment information were excluded.

All patients underwent a standardized diagnostic evaluation, including neurological examination by a board-certified neurologist, laboratory testing, electrocardiography, and vascular imaging with CT and/or MR angiography (Jung et al., 2024). Eligibility for EVT was determined by an interdisciplinary stroke team comprising experienced vascular neurologists and interventional neuroradiologists in accordance with institutional protocols and contemporary international guidelines (Jung et al., 2024; Powers and Rabinstein, 2019; Kennedy et al., 2016; Goyal et al., 2016; Sacks et al., 2018). All angiographic findings were independently reviewed by two experienced neuroradiologists.

Following EVT, patients were admitted to the stroke unit, intermediate care unit, or intensive care unit according to clinical need. Follow-up CT and/or MR imaging was routinely performed 24 h after EVT or earlier in cases of neurological deterioration.

The primary outcome was favorable functional outcome at 3 months, defined as a modified Rankin Scale (mRS) score of 0–2 (van Swieten et al., 1988). Secondary outcomes included excellent functional outcome (mRS 0–1), successful reperfusion (modified Thrombolysis in Cerebral Infarction [mTICI] score 2b−3), in-hospital mortality, 3-month mortality, symptomatic intracerebral hemorrhage (sICH), recurrent in-hospital ischemic stroke, and discharge destination.

Given the retrospective registry-based design, no formal *a priori* sample-size calculation was performed. The study sample comprised all consecutive patients fulfilling the predefined eligibility criteria during the study period.

Three-month functional outcome was assessed during an outpatient follow-up visit by a board-certified neurologist or by a structured telephone interview conducted by a trained study nurse. A standardized assessment procedure was used to promote consistency across follow-up modalities; however, formal inter-rater reliability testing was not performed.

Symptomatic intracerebral hemorrhage was defined as any intracranial hemorrhage associated with neurological deterioration of ≥4 points on the National Institutes of Health Stroke Scale (NIHSS) or resulting in death.

### Statistical analysis

All statistical analyses were performed using SPSS Statistics version 25.0 (IBM Corp., Armonk, NY, USA). Baseline characteristics, vascular risk factors, stroke etiology, treatment characteristics, workflow metrics, and outcomes were compared between women and men. Categorical variables are presented as frequencies and percentages and were compared using the χ^2^ test. Continuous variables are presented as median (interquartile range) or mean (standard deviation), as appropriate, and were compared using the independent-samples *t* test or the Mann–Whitney *U* test, depending on data distribution. All tests were two-sided, and a *p* value < 0.05 was considered statistically significant.

Multivariable binary logistic regression analyses were performed to evaluate the independent association between sex and successful reperfusion (mTICI 2b−3), favorable functional outcome at 3 months (mRS 0–2), excellent functional outcome at 3 months (mRS 0–1), and 3-month mortality. Covariates were selected *a priori* based on clinical relevance and previous literature while avoiding clinically overlapping variables. The final models included sex, age, NIHSS on admission, pre-stroke modified Rankin Scale, atrial fibrillation, coronary heart disease, chronic kidney disease, main occlusion site, bridging therapy, groin puncture-to-reperfusion time and calendar period. Sex was retained in all models as the exposure variable of interest. Groin-puncture-to-reperfusion time was not included in the successful reperfusion model, because it occurs downstream of that procedural outcome.

Additional effect-modification analyses were performed for successful reperfusion, favorable and excellent functional outcome at 3 months, and 3-month mortality by adding interaction terms between sex and age, stroke etiology, or bridging therapy separately to the respective multivariable models. Age was analyzed continuously per 10-year increase, stroke etiology as a multi-category variable using a global interaction test, and bridging therapy as direct EVT vs. IVT followed by EVT.

Adjusted odds ratios (aORs) with corresponding 95% confidence intervals (CIs) were reported. Missing data were not imputed, and complete-case analyses were performed.

### Ethics approval and consent to participate

The local part of the Swiss Stroke Registry was approved by the regional ethics committee (KEK Bern 2016-01905) for quality assurance and research purposes. Written informed consent was waived, and all patients were informed about the use of their medical data. In accordance with Swiss regulations, patients who declined the use of their data were excluded. The study was conducted in accordance with the Declaration of Helsinki and is reported in accordance with the STROBE guidelines.

## Results

## Baseline characteristics

The cohort included 581 patients treated during 2015–2017 and 1,183 treated during 2018–2023. Women accounted for 48.7 and 48.2% of patients, respectively.

A total of 1,179 patients were excluded: 1,018 did not undergo EVT, 145 had combined anterior and posterior circulation stroke, nine had no angiographically confirmed vessel occlusion, and seven had incomplete treatment data. The study cohort therefore comprised 1,764 patients with anterior circulation AIS treated with EVT (Figure 1).

**Figure 1:**
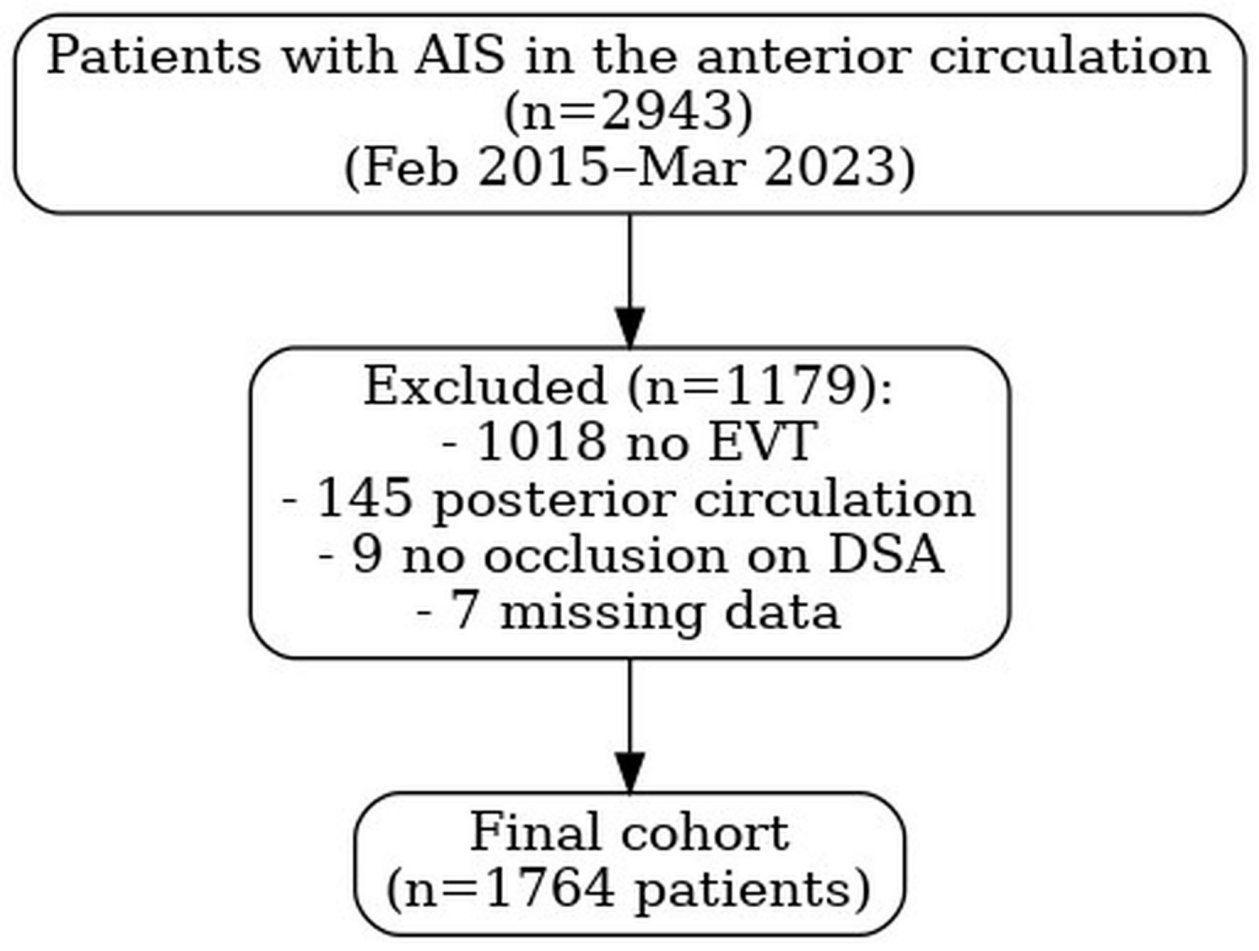
Flowchart of patients who met inclusion and exclusion criteria.

Of these, 911 (51.6%) were men and 853 (48.4%) were women (Table 1). Women were older than men (median age 79.4 vs. 73.3 years, *p* < 0.001) and more frequently had atrial fibrillation (43.3 vs. 35.7%, *p* = 0.001) and chronic kidney disease (26.7 vs. 20.9%, *p* = 0.004). In contrast, men more often had coronary heart disease (25.9 vs. 13.8%, *p* < 0.001), heart failure (6.9 vs. 4.0%, *p* = 0.007), diabetes mellitus (21.7 vs. 17.7%, *p* = 0.034), and were current smokers (25.0 vs. 14.9%, *p* < 0.001). The prevalence of arterial hypertension and peripheral artery disease did not differ significantly between the sexes.

**Table 1:** Baseline characteristics of the study cohort by sex.

Characteristic	Men (*N* = 911)	Women (*N* = 853)	Total (*N* = 1,764)	*P*-value
Calendar period				0.839
2015–2017	298 (32.7%)	283 (33.2%)	581 (32.9%)	
2018–2023	613 (67.3%)	570 (66.8%)	1,183 (67.1%)	
Age, median	73.3	79.4	76.2	**< 0.001**
NIHSS on admission	13 (0–42)	14 (0–42)	13 (0–42)	0.143
Independent prior stroke (mRS ≤ 2)	775 (85.1 %)	659 (77.3%)	1,434 (81.3%)	**< 0.001**
Atrial fibrillation	325 (35.7%)	369 (43.3%)	694 (39.3%)	**0.001**
Arterial hypertension	665 (73.0%)	655 (76.8%)	1,320 (74.8%)	0.067
Coronary heart disease	236 (25.9%)	118 (13.8%)	354 (20.1%)	**< 0.001**
Heart failure	63 (6.9%)	34 (4.0%)	97 (5.5%)	**0.007**
Diabetes mellitus	198 (21.7%)	151 (17.7%)	349 (19.8%)	**0.034**
Current smoking	228 (25.0%)	127 (14.9%)	355 (20.1%)	**< 0.001**
Chronic kidney disease	190 (20.9%)	228 (26.7%)	418 (23.7%)	**0.004**
Hypercholesterolaemia	614 (67.4%)	551 (64.0%)	1,165 (66.0%)	0.214
Peripheral artery disease	63 (6.9%)	52 (6.1%)	115 (6.5%)	0.486
Patients with pre stroke antihypertensives	548 (60.3%)	566 (66.5%)	1,114 (63.3%)	**0.007**
Patients with pre stoke statins	294 (32.3%)	211 (24.7%)	505 (28.6%)	**< 0.001**
Patients with pre stroke antithrombotics	866 (95.1%)	813 (95.3%)	1,679 (95.2%)	**0.022**
Stroke etiology				**< 0.001**
Cardioembolic stroke	356 (39.1%)	395 (46.3%)	751 (42.6%)	
Large artery atherosclerosis	165 (18.1%)	96 (11.3%)	261 (14.8%)	
More than one etiology	52 (5.7%)	45 (5.7%)	97 (5.5%)	
Other causes	40 (4.4%)	37 (4.4%)	77 (4.4%)	
Cervical artery dissection	35 (3.8%)	11 (1.3%)	46 (2.6%)	
Unclear, complete evaluation	26 (2.9%)	27 (2.9%)	53 (3.0%)	
Unclear, incomplete evaluation	237 (26.0%)	242 (28.4%)	479 (27.2%)	
Wake up stroke	181 (19.9%)	143 (16.8%)	324 (18.4%)	0.092
Admission systolic blood pressure (mmHg) (median)	154	159	156	**0.012**
Admission diastolic blood pressure (mmHg) (median)	83	82	82	0.496
Stroke patients transfer from other hospital	381 (41.8%)	337 (39.5%)	718 (40.7%)	0.323
IVT administered in other hospital	159 (17.5%)	121 (14.2%)	280 (15.9%)	0.061
Admission HbA1C (median) (%)	5.8	5.7	5.75	0.077
LDL (median) (mmol/L)	2.3	2.56	2.4	**< 0.001**
Triglycerides (median) (mmol/L)	1.28	1.29	1.28	**0.034**
Hemoglobin (median) (g/L)	139	127	132	**< 0.001**
C-reactive protein (median) (CRP, mg/L)	4.0	4.0	4.0	0.708
Pre-stroke therapies				
Antihypertensive	548 (60.3%)	566 (66.5%)	1,114 (63.3%)	**0.007**
Statin treatment	294 (32.3%)	211 (24.7%)	505 (28.6%)	**< 0.001**
Antithrombotic treatment	866 (95.1%)	813 (95.3%)	1,679 (95.2%)	**0.022**

Bold values indicate statistical significance (*p* < 0.05).

Cardioembolic stroke was more frequent in women (46.3 vs. 39.1%), whereas large artery atherosclerosis predominated in men (18.1 vs. 11.3%; both *p* < 0.001).

The distribution of vessel occlusion sites was similar between women and men (*p* = 0.223; Table 2).

**Table 2:** Main occlusion site–characteristics by sex.

**Main occlusion site**	Men	Women	Overall	*P*-value
ICA-I	50 (5.5%)	39 (4.6%)	89 (5.0%)	0.223
ICA-T	121 (13.3%)	143 (16.8%)	264 (15.0%)	
ICA-external	20 (2.2%)	17 (2.0%)	37 (2.1%)	
Proximal M1segment	240 (26.3%)	234 (27.4%)	474 (26.9%)	
Distal M1 segment	169 (18.6%)	172 (20.2%)	341 (19.3%)	
M2 segment	286 (31.4%)	232 (27.2%)	518 (29.4%)	
M3	15 (1.6%)	8 (0.9%)	23 (1.3%)	
ACA	9 (1.0%)	6 (0.7%)	15 (0.9%)	
CCA	0 (0%)	1 (0.1%)	1 (0.1%)	
Others	1 (0.1%)	1 (0.1%)	2 (0.2%)	

ICA-I occlusion, non-terminal intracranial internal carotid artery occlusion; ICA-T, terminal intracranial internal carotid artery occlusion; M1 M2 M3 segments MCA, middle cerebral artery; CCA, common carotid artery; ACA, anterior cerebral artery; PCA, posterior cerebral artery.

Workflow metrics were similar between women and men (Table 3). Median times from last known well to hospital arrival, door to imaging, imaging to groin puncture, imaging to intravenous thrombolysis, and door to groin puncture did not differ significantly. Women had a slightly shorter median groin puncture-to-reperfusion time than men (40.2 vs. 42.0 min, *p* = 0.038).

**Table 3:** Workflow metrics, endovascular treatment characteristics and procedural outcomes according to sex.

Variable	Men (*n* = 911)	Women (*n* = 853)	Overall (*n* = 1,764)	*P*-value
**Workflow metrics, min (median, range)**				
Last known well → Hospital arrival	150 (34–1,205)	150 (32–1,269)	150 (32–1,269)	0.947
Door → Imaging	18 (6–60)	18 (5–60)	18 (5–60)	0.095
Imaging → Groin puncture	55 (17–178)	55 (18–180)	55 (17–180)	0.993
Imaging → IV thrombolysis^*^	18.0 (2–50)	18.2 (7–49)	18.0 (2–50)	0.483
Door → Groin puncture	75.2 (33–201)	76.3 (34–210)	76.0 (33–210)	0.649
Groin puncture → Reperfusion	42.0 (15–120)	40.2 (15–120)	40.8 (15–120)	**0.038**
**Treatment approach**				
Bridging therapy	372 (40.8)	296 (34.7)	668 (37.9)	**0.008**
Intracranial stenting	32 (3.5)	13 (1.5)	45 (2.6)	**0.008**
Extracranial stenting	191 (21.0)	81 (9.5)	272 (15.4)	**< 0.001**
**Type of anesthesia**				
General anesthesia	809 (88.8)	734 (86.0)	1,543 (87.5)	
Conscious sedation	102 (11.2)	119 (14.0)	221 (12.5)	0.081
Devices used				
Stent retriever	840 (92.2)	799 (93.8)	1,639 (92.9)	0.231
Distal retriever	106 (11.6)	93 (10.9)	199 (11.3)	0.627
Aspiration	421 (46.2)	384 (45.0)	805 (45.6)	0.614
Balloon angioplasty	45 (4.9)	41 (4.8)	86 (4.9)	0.897
Intra-arterial thrombolytic drug	40 (4.4)	25 (2.9)	65 (3.7)	0.104
Number of stent retriever passes	1 (1–7)	1 (1–7)	1 (1–7)	0.104
Procedural outcomes				
Successful reperfusion (mTICI 2b−3)	798 (87.6)	723 (84.8)	1,521 (86.2)	0.084
EVT complications	231 (25.4)	207 (24.3)	438 (24.8)	0.597

* Data are presented as median (minimum–maximum). Imaging → IVT analysis includes only patients treated with IVT (male n = 372; female n = 296).

Bold values indicate statistical significance (*p* < 0.05).

Treatment strategies differed between the sexes. Men more frequently received bridging therapy (40.8 vs. 34.7%, *p* = 0.008), whereas intracranial (3.5 vs. 1.5%, *p* = 0.008) and extracranial stenting (21.0 vs. 9.5%, *p* < 0.001) were also performed more often in men. No significant sex differences were observed regarding anesthesia, thrombectomy technique, device selection, number of stent retriever passes, intra-arterial thrombolysis, balloon angioplasty, or procedural complications.

## Outcomes

Successful reperfusion (mTICI 2b−3) was achieved in 86.2% of patients and did not differ significantly between women and men (84.8 vs. 87.6%, *p* = 0.084; Table 4). Similarly, the rates of symptomatic intracerebral hemorrhage (5.2 vs. 6.7%, *p* = 0.173) and recurrent in-hospital ischemic stroke (6.9 vs. 6.5%, *p* = 0.711) were similar between the sexes.

**Table 4:** Sex differences in stroke outcomes.

Outcome	Men	Women	Total	*P*-value
Favorable outcome at 3 months (mRS ≤ 2)	426 (47.6%)	313 (37.5%)	739 (42.7%)	**< 0.001**
Excellent outcome at 3 months (mRS ≤ 1)	281 (31.4%)	214 (25.6%)	495 (28.6%)	**0.008**
Mortality at discharge	95 (10.4%)	112 (13.1%)	207 (11.7%)	0.078
Mortality at 3 months	227 (24.9%)	272 (31.9%)	499 (28.3%)	**0.001**
sICH	61 (6.7%)	44 (5.2%)	105 (6.0%)	0.173
New in-hospital isachemic stroke	59 (6.5%)	59 (6.9%)	118 (6.7%)	0.711
Successful reperfusion (TICI 2b/2c/3)	798 (87.6%)	723 (84.8%)	1,521 (86.2%)	0.084
Discharge destination				**0.002**
Home	121 (13.3%)	71 (8.3%)	192 (10.9%)	
Neurorehabilitation	198 (21.7%)	165 (19.4%)	363 (20.6%)	
Other acute care hospital	484 (53.1%)	483 (56.7%)	967 (54.8%)	
Nursing home	13 (1.4%)	21 (2.5%)	14 (1.9%)	
Death at discharge	95 (10.4%)	112 (13.1%)	207 (11.7%)	

sICH symptomatic, symptomatic intracerebral hemorrhage; TICI, Thrombolysis in Cerebral Infarction scale.

Bold values indicate statistical significance (*p* < 0.05).

Favorable functional outcome at 3 months was achieved by 235 patients (40.4%) in 2015–2017 and 504 patients (43.9%) in 2018–2023. Despite similar reperfusion rates, functional outcome differed between the sexes. At 3 months, men more frequently achieved favorable functional outcome (mRS 0–2: 47.6 vs. 37.5%, *p* < 0.001) and excellent functional outcome (mRS 0–1: 31.4 vs. 25.6%, *p* = 0.008). Although in-hospital mortality was similar (10.4 vs. 13.1%, *p* = 0.078), women had a higher 3-month mortality than men (31.9 vs. 24.9%, *p* = 0.001).

Discharge destination also differed between the sexes (*p* = 0.002). Men were more frequently discharged home or to inpatient rehabilitation, whereas women were more often transferred to another acute-care hospital or discharged to a nursing home (Table 4).

## Multivariable analysis

Female sex was not independently associated with successful reperfusion (aOR 0.79; 95% CI, 0.60–1.06; *p* = 0.117), favorable functional outcome (aOR 0.88; 95%CI, 0.69–1.11; *p* = 0.267), excellent functional outcome (aOR 0.93; 95%CI, 0.73–1.19; *p* = 0.584), or 3-month mortality (aOR 1.06; 95%CI, 0.83–1.35; *p* = 0.647). Successful reperfusion was additionally associated with occlusion site, calendar period, and pre-stroke mRS. Younger age, lower admission NIHSS and pre-stroke mRS, and shorter groin-puncture-to-reperfusion time were associated with better functional outcomes. Bridging therapy was associated with favorable functional outcome. Older age, higher admission NIHSS and pre-stroke mRS, and chronic kidney disease were associated with higher 3-month mortality, whereas bridging therapy and treatment during 2018–2023 were associated with lower mortality (Figure 2).

**Figure 2:**
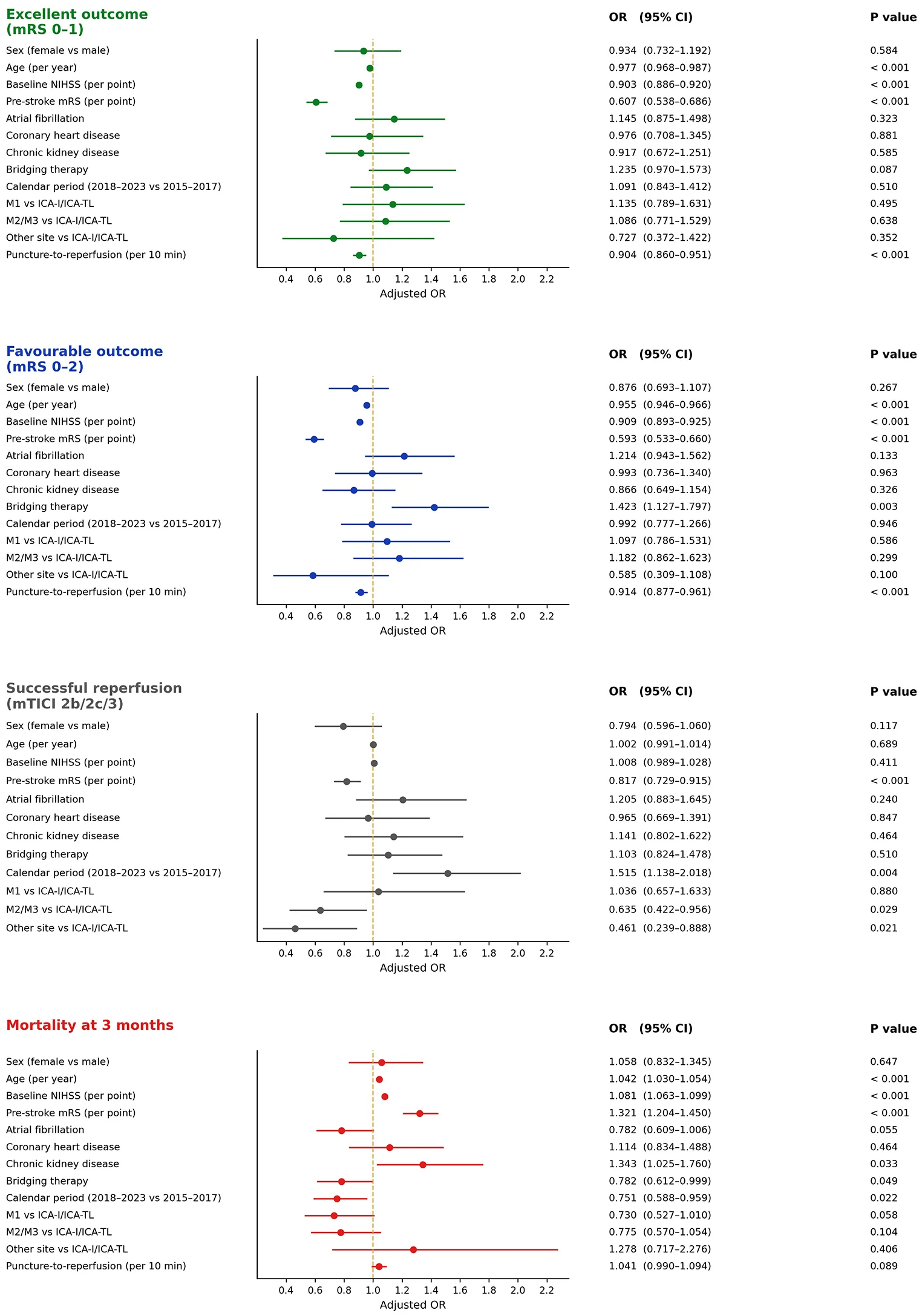
Multivariable logistic regression analysis of predictors of clinical outcomes, successful reperfusion, and 3-month mortality. Adjusted odds ratios (ORs) with 95% confidence intervals (CIs) are shown. The x-axis represents the adjusted OR, and the y-axis represents the predictor factors included in the multivariable models. M1, M2, and M3 indicate the respective middle cerebral artery occlusion segments, and ICA-I/ICA-TL is the reference category for Internal carotid artery occlusion site. Puncture-to-reperfusion time is expressed per 10-min increase and was not included in the successful-reperfusion model.

A sex-by-age interaction was observed for favorable functional outcome at 3 months (*p* for interaction = 0.006; ratio of odds ratios per 10-year increase, 1.30; 95%CI, 1.08–1.57). No sex-by-age interactions were found for the other outcomes (all *p* for interaction ≥0.069). There was no evidence of effect modification by stroke etiology or bridging thrombolysis (all *p* for interaction ≥0.058).

## Discussion

Our study evaluated sex-specific differences in vascular risk factors, stroke etiology, treatment characteristics, and outcomes in a large contemporary cohort of patients with anterior circulation acute ischemic stroke undergoing EVT. Women and men showed distinct vascular risk profiles, with atrial fibrillation and chronic kidney disease predominating in women and coronary heart disease, heart failure, diabetes mellitus, and smoking in men. Consistent with these differences in vascular risk profiles, cardioembolic stroke predominated in women, whereas large artery atherosclerosis was more frequent in men.

Despite these baseline differences, procedural success was similar between women and men. Although women had less favorable unadjusted functional outcomes and higher 3-month mortality, these differences were no longer evident after adjustment for baseline characteristics. After adjustment, sex was not independently associated with outcomes overall, although its association with favorable functional outcome varied according to age.

## Sex-specific differences in vascular risk factors and stroke etiology

Our findings are consistent with previous reports demonstrating distinct sex-specific differences in vascular risk factors and stroke etiology in acute ischemic stroke (Ospel et al., 2023; Appelros et al., 2009; Petrea et al., 2009; Niewada et al., 2005; Peters et al., 2013; Toyoda and Ninomiya, 2014; Madsen et al., 2019b; Yang et al., 2023). Women were considerably older and more frequently had atrial fibrillation and chronic kidney disease, whereas men more commonly had coronary heart disease, heart failure, diabetes mellitus, and current smoking. This pattern is pathophysiologically plausible: older age and atrial fibrillation in women favor atrial thrombus formation and cardioembolism, whereas the higher prevalence of coronary heart disease, diabetes mellitus, and smoking in men indicates a greater systemic atherosclerotic burden, consistent with the predominance of large-artery atherosclerosis.

The higher prevalence of chronic kidney disease among women may partly reflect their older age and greater burden of vascular comorbidity. Chronic kidney disease is an established risk factor for cerebrovascular disease through chronic inflammation, endothelial dysfunction, and a prothrombotic state. Heart failure, which was more common in men, is closely linked to coronary heart disease and has likewise been associated with an increased risk of cerebrovascular events (Toyoda and Ninomiya, 2014; Yang et al., 2023).

Although the prevalence of arterial hypertension did not differ significantly between the sexes in our cohort, previous studies suggest that hypertension may confer a greater relative risk of stroke in women than in men, even after adjustment for antihypertensive treatment (Peters et al., 2020; Niewada et al., 2005; Peters et al., 2013; Madsen et al., 2019b).

Overall, these findings highlight substantial sex-specific differences in vascular risk profiles and stroke mechanisms that should be considered when developing targeted stroke prevention strategies.

Variation between previous studies may partly reflect differences in age distribution, vascular risk profiles, definitions of stroke etiology, and adjustment for potential confounders. In our cohort, the observed aetiological differences closely paralleled the corresponding sex-specific risk-factor patterns, although the underlying mechanisms were not directly assessed.

Overall, these findings highlight sex-specific differences in vascular risk profiles and stroke mechanisms that may inform targeted stroke-prevention strategies.

## Sex-specific differences in workflow metrics and treatment approach

Workflow metrics were broadly similar between women and men, except for a slightly shorter groin-puncture-to-reperfusion time in women that was unlikely to be clinically relevant. These findings contrast with previous studies reporting longer prehospital delays and lower rates of timely reperfusion therapy in women, which have been associated with poorer clinical outcomes (Bushnell C. et al., 2018; Grimes et al., 2024; Ospel et al., 2023; Demel et al., 2022; Mainz et al., 2020; Uchida et al., 2019; Otite et al., 2021; van der Meij et al., 2024). The absence of such disparities in our cohort may reflect well-established regional stroke pathways, standardized emergency medical services triage, and equitable access to comprehensive stroke care.

Treatment strategies differed between the sexes. Men were more likely to receive bridging therapy and to undergo intracranial and extracranial stenting, most likely reflecting the higher prevalence of large artery atherosclerosis. In contrast, women more commonly presented with cardioembolic stroke, which may reduce the need for adjunctive endovascular procedures. These findings are consistent with previous reports describing lower rates of atherosclerosis and sex-related differences in vascular anatomy, including smaller vessel diameters and greater vessel tortuosity in women (Casetta et al., 2022).

Importantly, these procedural differences did not translate into differences in reperfusion success, thrombectomy technique, anesthesia strategy, or procedural complications.

Overall, these findings indicate that contemporary EVT can be performed with similar technical success and procedural safety in both women and men despite differences in stroke etiology and treatment approach. Thus, the crude outcome differences are unlikely to be explained by sex-related disparities in procedural access or success.

## Sex-specific differences in outcomes

Despite similar reperfusion rates, women had less favorable functional outcomes, and higher 3-month mortality in the unadjusted analyses. These findings are consistent with several previous studies reporting worse crude outcomes among women following EVT (de Ridder et al., 2016; Dmytriw et al., 2021; Chalos et al., 2019; Madsen et al., 2019a; Dula et al., 2020; Sheth et al., 2019; Ouyang et al., 2023; Fifi et al., 2023; Single et al., 2025). However, more recent registry studies and meta-analyses have shown that these differences are largely attenuated after adjustment for age, baseline stroke severity, pre-stroke disability, and other prognostic factors, with some studies even suggesting a modest benefit of EVT in women (Chalos et al., 2019; Ouyang et al., 2023; Fifi et al., 2023; Single et al., 2025; Noseda et al., 2025; Ospel et al., 2022). The heterogeneity across previous studies may reflect differences in age distribution, pre-stroke disability, stroke severity, patient selection, included vascular territories, EVT treatment era, and covariate adjustment. Our findings are consistent with this growing body of evidence. Although women experienced worse unadjusted outcomes, female sex was not independently associated with functional recovery or 3-month mortality after multivariable adjustment.

The poorer crude outcomes in women are consistent with their older age and greater pre-stroke disability, both of which were independently associated with outcomes and may indicate reduced functional reserve and recovery potential. Similar reperfusion rates between women and men argue against lower technical efficacy of EVT as the principal explanation.

Women were less frequently discharged home or to inpatient rehabilitation. However, because frailty, living situation, socioeconomic status, social support, and access to rehabilitation were not recorded, the reasons for this difference and its potential contribution to functional outcome remain uncertain (Single et al., 2025; Niewada et al., 2005).

Taken together, sex was not independently associated with outcomes overall, although the sex-by-age interaction suggests possible age-related heterogeneity in favorable functional outcome. Age, pre-stroke disability, stroke severity, and comorbidity burden were more strongly associated with outcomes than sex itself. The observed sex-by-age interaction was exploratory and requires confirmation in independent cohorts.

## Clinical implications

Our findings support current guideline recommendations that sex alone should not influence patient selection for EVT. Instead, treatment decisions should be guided by established clinical and imaging criteria, irrespective of sex (Ospel et al., 2022).

Second, the marked differences in vascular risk profiles between women and men highlight the importance of sex-specific prevention strategies. Greater emphasis on atrial fibrillation detection and management may be particularly relevant in women, whereas aggressive control of atherosclerotic risk factors, including smoking cessation, diabetes management, and lipid lowering, remains crucial in men.

The less favorable unadjusted outcomes observed in women were consistent with their older age and greater pre-stroke disability, both of which were independently associated with outcomes. Sex was not independently associated with outcomes overall, although its association with favorable functional outcome varied by age. Whether unmeasured factors such as frailty, living situation, socioeconomic status, social support, or access to rehabilitation contribute further remains to be established. These findings support consideration of baseline functional status and modifiable pre-stroke risk factors but do not provide evidence for sex-specific post-stroke interventions.

## Future directions

Future multicenter studies with predefined sex-specific analyses are needed to validate the observed sex-by-age interaction and further clarify differences in outcomes after EVT. Future research should distinguish sex from gender-related factors and incorporate measures of frailty, socioeconomic status, living situation, social support, and access to rehabilitation. In addition, further investigation of cerebrovascular physiology, clot characteristics, and recovery mechanisms may improve our understanding of sex-specific differences and support more individualized approaches to stroke care. Adequate representation of women and routine sex-specific analyses should become standard in future clinical trials (Ospel et al., 2022).

## Strengths

Our study has several strengths. It represents one of the largest contemporary single-center cohorts of patients with anterior circulation acute ischemic stroke treated with EVT using standardized diagnostic and therapeutic pathways. The prospectively maintained stroke registry ensured comprehensive and high-quality data collection. Unlike many previous studies, we focused exclusively on anterior circulation stroke and comprehensively evaluated sex-specific differences in treatment characteristics and outcomes after EVT. Furthermore, multivariable analyses enabled us to determine whether observed sex differences remained independently associated with outcomes after adjustment for clinically relevant baseline characteristics.

## Limitations

Several limitations should be acknowledged. First, the retrospective single-center design may limit the generalizability of our findings, although the large sample size and prospectively maintained registry strengthen the robustness of our results. Second, the study period spanned 8 years, during which advances in EVT techniques, devices, and treatment recommendations may have influenced patient selection and clinical outcomes. Third, potential explanatory factors, including clot composition, vascular anatomy, hormonal status, frailty, and physiological reserve, were not measured; therefore, the proposed mechanisms remain hypothesis-generating. Residual confounding cannot be excluded despite multivariable adjustment. The registry captured sex as documented in the medical record, whereas gender identity and gender-related social factors were not assessed. Data on living situation, socioeconomic status, social support, and access to rehabilitation were unavailable; therefore, their potential contribution to functional outcome remains uncertain. Finally, the effect-modification analyses were exploratory, multiple interaction tests were performed without adjustment for multiple testing, and some stroke-etiology subgroups may have had limited statistical power.

## Conclusion

In this large contemporary cohort of patients with anterior circulation AIS treated with EVT, women and men differed substantially in vascular risk profiles and stroke etiology but achieved similar reperfusion rates. Although women had worse unadjusted outcomes, sex was not independently associated with outcomes overall; however, its association with favorable functional outcome varied according to age. These findings support equitable access to EVT irrespective of sex and highlight the importance of individualized patient management based on clinical characteristics rather than sex alone.

## Data Availability

The raw data supporting the conclusions of this article will be made available by the authors, without undue reservation.
